# Gene-environment interactions and the effect on obesity risk in low and middle-income countries: a scoping review

**DOI:** 10.3389/fendo.2023.1230445

**Published:** 2023-08-18

**Authors:** Sophia L. Pledger, Fariba Ahmadizar

**Affiliations:** ^1^Department of Epidemiology and Global Health, Julius Global Health, University Medical Center Utrecht, Utrecht, Netherlands; ^2^Department of Data Science and Biostatistics, Julius Global Health, University Medical Center Utrecht, Utrecht, Netherlands

**Keywords:** genes, single nucleotide polymorphism, gene-environment interaction, obesity, low and middle-income countries, review

## Abstract

**Background:**

Obesity represents a major and preventable global health challenge as a complex disease and a modifiable risk factor for developing other non-communicable diseases. In recent years, obesity prevalence has risen more rapidly in low- and middle-income countries (LMICs) compared to high-income countries (HICs). Obesity traits are shown to be modulated by an interplay of genetic and environmental factors such as unhealthy diet and physical inactivity in studies from HICs focused on populations of European descent; however, genetic heterogeneity and environmental differences prevent the generalisation of study results to LMICs. Primary research investigating gene-environment interactions (GxE) on obesity in LMICs is limited but expanding. Synthesis of current research would provide an overview of the interactions between genetic variants and environmental factors that underlie the obesity epidemic and identify knowledge gaps for future studies.

**Methods:**

Three databases were searched systematically using a combination of keywords such as “genes”, “obesity”, “LMIC”, “diet”, and “physical activity” to find all relevant observational studies published before November 2022.

**Results:**

Eighteen of the 1,373 articles met the inclusion criteria, of which one was a genome-wide association study (GWAS), thirteen used a candidate gene approach, and five were assigned as genetic risk score studies. Statistically significant findings were reported for 12 individual SNPs; however, most studies were small-scale and without replication.

**Conclusion:**

Although the results suggest significant GxE interactions on obesity in LMICs, updated robust statistical techniques with more precise and standardised exposure and outcome measurements are necessary for translatable results. Future research should focus on improved quality replication efforts, emphasising large-scale and long-term longitudinal study designs using multi-ethnic GWAS.

## Introduction

Obesity is a major public health concern worldwide, with 39% of adults over 18 classified as overweight and 13% obese, according to the most recent global estimates by the Word Health Organisation (WHO) (1). Previously considered a disease primarily affecting high-income counties (HICs), obesity is rapidly rising in developing countries with emerging economies. These low- and middle-income countries (LMICs) are now home to 62% of the world’s overweight or obese population (2) and make up the top 10 countries with the largest average annual increase in obesity prevalence worldwide (3). In recent decades, LMICs have faced an epidemiological transition, characterised by a shift in the main drivers of mortality and morbidity from communicable diseases to non-communicable diseases (NCDs), such as cardiovascular disease, type 2 diabetes and cancer. As both a major metabolic risk factor for NCD development and a disease by itself, obesity represents a significant epidemiological burden (4). While the prevention and treatment of obesity is a primary target within global health systems, it poses significant challenges thanks to its complexity as a disease and the contribution of a multifaceted interplay of variables which underwrite its development.

Obesity, defined by the WHO as a body mass index (BMI) of ≥ 30 kg/m2, is determined by a long-term positive imbalance in energy intake versus energy expenditure, driven by an unhealthy diet and reduced physical activity (1, 5). Changes in global trade, dietary patterns, and declining physical activity have exposed people living in developing countries to increasingly obesogenic environments (2). The nutrition transition faced by LMICs has been a significant contributor to the obesity epidemic, characterised by a shift from traditional dietary habits to increased consumption of energy-dense, nutrient-poor ultra-processed foods and beverages (6). Rapid urbanisation and within-country rural-to-urban migration have also led to decreased manual labour and active transportation and increased sedentary behaviours (7, 8). Genetics has also been shown to play a substantial role in an individual’s susceptibility to obesity, with obesity heritability estimated to be between 40-70% (9). The advent of genome-wide association studies (GWAS) accelerated the discovery of obesity-related genetic loci and causative single nucleotide polymorphisms (SNPs) but focussed on adult populations of European ancestry (10, 11). The fat mass and obesity-associated (FTO) locus was the first obesity-related GWAS-identified locus and remains the most highly significant and robustly replicated (11, 12). The landmark 2007 European study initially found that per risk allele in the FTO SNP rs9939609, there was a 1.32-fold increased odds of obesity (13). The effect of FTO SNPs on obesity risk and the prevalence of FTO risk alleles has since been shown to vary across different ethnic populations (14), with the risk of obesity per risk allele increasing 1.25-fold in Asians and 1.15 in Indians (15, 16). Since the discovery of FTO, an additional 1,100 independent genome-wide significant loci have been identified; however, these combined explain only 6% of inter-individual obesity variation (17).

As both a modifiable risk factor and a complex multifactorial condition, obesity results from an interplay of genetic, lifestyle and environmental factors (12). In parallel with GWAS, the number of studies analysing gene-environment interactions (GxE) on obesity risk has increased exponentially over the last decade; however, these have focussed primarily on European populations living in resource-rich settings (18). Recent research from HICs has presented evidence that an individual’s genetic susceptibility to obesity can be magnified or mitigated in response to environmental factors, such as physical activity (19), alcohol consumption (20), smoking (21), diet (22) and sleep (23). The generalisability of findings from these studies to developing countries is restricted due to the genetic heterogeneity found in different populations and ethnic groups and differences in obesogenic environmental exposure (24). So far, observational research focussing on people from LMICs is limited but expanding. Synthesis of population-based studies specifically investigating GxE on obesity in LMICs could provide a more comprehensive understanding of this cause-effect relationship in varied ethnic groups and potentially translate into tailored region-specific lifestyle intervention strategies to combat the global obesity epidemic. Therefore, we reviewed and discussed the results of population-based studies from LMICs investigating gene-lifestyle interactions and their effect on obesity.

## Materials and methods

This study followed the Transparent Reporting of Systematic Reviews and Meta-analyses (PRISMA) guidelines (25).

### Eligibility criteria

The study inclusion and exclusion criteria for this review were specified using the PECOS elements, as defined in **Table 1**. All observational research articles investigating GxE on obesity risk in adult human populations from LMICs were included. Articles were also excluded if they focussed on specific populations, e.g., only women or participants with comorbidities such as cancer, cardiovascular or renal diseases, to reduce concerns for disease labelling bias.

**Table 1:** PECOS criteria for inclusion and exclusion of studies.

	Inclusion criteria	Exclusion criteria
Population	Human participants >18 years old All genders and ethnicities Populations in LMIC’s	Animal studies Populations from HIC’s Studies only including men or women Paediatric studies Participants with concomitant disease such as cancer, CVD disease, hypertension, renal disease, neurological diseases. Pregnant or breastfeeding women
Exposure	Studies investigating gene-environment interactions on obesity risk. Exposures were defined as a combination of genetic susceptibility (e.g. genome-wide association studies, polygenic risk scores, genetic risk scores, single nucleotide polymorphisms, epigenetics, and methylation) and lifestyle and environmental factors (e.g. diet, smoking, physical activity).	Studies that did not include both exposures.
Comparison	Any or no comparators.	–
Outcome	Obesity (as a disease or risk factor) as a binary outcome, or continuous outcomes such as weight-related anthropometric measurements (BMI, weight, waist circumference, waist-to-hip ratio) or body composition indices (body fat percentage).	Studies which did not report the outcome of interest (namely studies only reporting type 2 diabetes).
Study Design	Observational studies, including case-control, cohort, or cross-sectional studies.	Randomised, non-randomised or placebo-controlled trials.

BMI; body mass index, CVD; cardiovascular disease, HIC; high-income countries, LMIC; low- and middle-income countries.

### Search strategy

Three electronic databases, PubMed, Embase and Scopus, were systematically searched to identify observational studies relevant to our research question and published before November 2022. The search strategy was subdivided into three main groupings: studies in LMICs, gene-environment interactions, and weight-related outcomes. To define LMICs, articles were searched by title and abstract for keywords such as ‘low, middle income’, ‘developing country’ and ‘low resource’. To further increase capture, the names of countries and geographical areas were included in the search syntax according to the World Bank 2022 country classifications (26). To include studies on gene-environment interactions, keywords were used such as ‘gene-lifestyle’ or ‘GxE’ or terms relating to genetic susceptibility such as ‘polygenic risk score’, ‘single nucleotide polymorphism’ and ‘epigenetic’ in combination with environmental exposure such as ‘diet’ or ‘physical activity’. Obesity and all other weight-related anthropometric measurements such as ‘BMI’, ‘waist circumference’, ‘body fat percentage’, and ‘waist-hip ratio’ were included in the final group. Animal, paediatric and intervention studies were excluded. No filters based on language or publication date were applied. Details of the search strategies developed for each database can be found in **Supplementary Table 1.**

### Data collection and extraction

Titles and abstracts of all articles identified *via* database searches were screened based on the eligibility criteria previously detailed using Endnote (v20.4.1) (27). Full-text articles were assessed using Rayyan, with all reasons for exclusion documented (28). Data extraction was also performed using a standardised form with the software programme Microsoft Excel 2016. For each included study, the following information was extracted:

• First author, year of study, year of publication, country of coverage, study objectives, and study design.
• Sample size, distribution of study population characteristics (e.g., BMI, age, gender), obesity definition, environmental or lifestyle exposure, genetic exposure (e.g., gene or SNP of interest) and type of genetic analysis.
• Primary and secondary results (e.g., β coefficient or odds ratio where possible) and overall conclusion.

## Results

**Figure 1:**
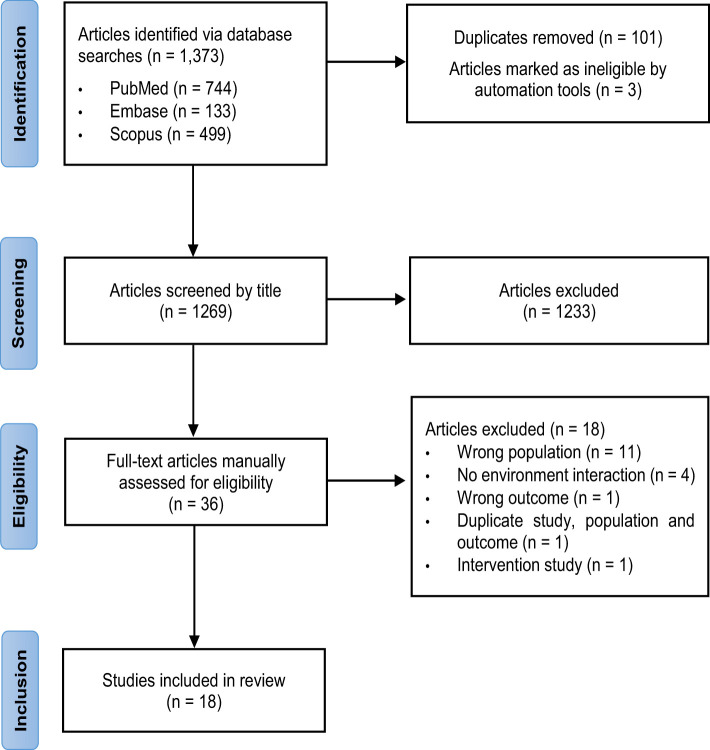
PRISMA flow chart for the selection of studies.

A total of 1,373 articles were identified, of which 744 were from PubMed, 133 from Embase and 499 from Scopus. After removing 101 duplicate studies and 3 ineligible studies, titles and abstracts of 1,269 articles were screened, and 1,233 irrelevant studies were excluded. Full texts were reviewed for 36 articles, of which 18 were excluded. Of these, 11 reported on populations which did not meet the inclusion or exclusion criteria, 4 did not include an environmental interaction, 1 reported an irrelevant outcome, and 1 incorporated an interventional design. Two studies by the same authors reported duplicate populations, studies and outcomes but differed by cross-sectional versus longitudinal analyses. The cross-sectional study was excluded to ensure the strength of evidence was not overestimated, and the most recent longitudinal study was included. In total, 18 studies met the PECOS criteria and were included in this review (29–46). **Figure 1** shows the PRISMA flow chart for the selection of studies.

### Characteristics of included studies

#### Populations and study designs

A summary of the key features of the 18 included studies is presented in **Tables 2**, **3**. In short, five studies were conducted in South Asia (Pakistan, Sri Lanka and India), three studies in South-East Asia (Indonesia), four studies in West Asia (Iran and Turkey), five studies in China, and one study in Ghana. Studies from China were assessed to ensure that study populations did not focus on very high-income regions. Across all studies, ages ranged between 18 to 90 years, and sample sizes ranged from 71 to 14,131 participants. Publication time ranged from 2012-2022. Most studies were cross-sectional (n = 9) or case-control (n = 7) by design, with only two cohort studies examining longitudinal associations.

**Table 2:** Summary of included observational studies investigating gene-environment interactions on obesity.

AUTHORS (YEAR)	STUDY COUNTRY/ DESIGN	STUDY OBJECTIVES	SAMPLE SIZE (FEMALE %; AGE DISTRIBUTION; BMI DISTRIBUTION)	OBESITY DEFINITION	LIFESTYLE/ ENVIRONMENTAL FACTORS	GENETIC APPROACH	PRIMARY RESULTS	SECONDARY RESULTS	CONCLUSION
**Muhammad et al. (42)**	Indonesia	Assess the role of *UCP2* gene variation on energy intake, PA and changes in adiposity	323 (50.8%; 42.8 ± 9.7; 25.1 ± 5.0)	–	Dietary intake (total energy, protein, fat and carbohydrate intake) and total PA (MET-min/week)	Candidate genes	Changes in body weight and %BF were positively associated with total energy intake. *UCP2* gene variation was not associated with changes in energy intake, dietary composition or PA	Energy intake was positively correlated with body weight and %BF changes and PA was negatively correlated with changes in WHR for *UCP2* GG genotypes but not AA or GA genotypes.	*UCP2*-866G/A GG genotypes are more susceptible to the association between energy intake and adiposity
	Cohort					*UCP2* −866G/A			
**Hosseini-Esfahani et al. (34)**	Iran	Explore the effect of dietary patterns on *FTO* SNPs and their effect on BMI and WC change.	4292 (56.8%; M 42.6 ± 14, F 40.4 ± 13)	–	Healthy (high levels of vegetables, fish, poultry, legumes, whole grains ) and Western dietary patterns (high intake of fast food, sweets, sugar and red meat)	Weighted GRS of 6 SNPs (*FTO* rs1421085, rs1121980, rs17817449, rs8050136, rs9939973 and rs3751812	High Western dietary pattern score was associated with 2-fold higher BMI for carriers of risk alleles rs1121980, rs1421085, rs8050136, rs1781799 and rs3751812	Significant interaction on high Western dietary pattern score and high GRS group compared with low GRS group on BMI increase (mean BMI change: 1.04±0.34 vs 2.26±0.36).	Western dietary patterns increase the association of *FTO* SNPs genetic susceptibility with BMI or WC increase.
	Cohort		No information on overall BMI distribution						
**Wei et al. (44)**	China	Assess the relationship between 3 *MC4R* SNPs, and their interaction with environmental factors on obesity	Cases: 858 (61.5%; 56.3 ± 14.3; 27.6 ± 3.1)	BMI ≥ 25 kg/m^2^	Smoking and alcohol drinking status	Candidate genes	All *MC4R* genotypes and minor allele frequencies were significantly different in obesity and control groups (*P*<0.05)	rs12970134 GA/AA genotypes and WC (≥90cm/≥80cm M/F) had higher obesity risk than GG genotypes and lower WC. No significant gene-smoking or -drinking interaction	MC4R SNPs were associated with the risk of obesity, but there was no significant gene-environment interactions.
	Case-control		Controls: 978 (47.3%; 55.4 ± 15.3; 20.0 ± 1.5)			*MC4R* rs17782313, rs476828 and rs12970134			
**Sun et al. (41)**	China	Investigate if olfactory pathway genes are related to obesity, and any interaction effects of smoking, alcohol drinking and PA	Cases: 301 (61.1%; 53.5 ± 11.1; ≥ 28 kg/m^2^)	BMI ≥28 kg/m^2^ AND	Smoking (current, non-smokers), alcohol and PA (inactive, moderate, vigorous)	Candidate genes	*OR4D1*, and *OR52K1* gene scores were positively correlated with obesity, and *OR2L8* and *CALML3* gene scores were negatively correlated with obesity.	Higher *OR4D1* gene score smokers were at a greater risk of obesity (OR = 2.67 [CI = 1.35, 5.30]), while high *CALML3* gene score smokers had a lower risk of obesity (OR= 0.25 [CI = 0.10, 0.62]).	Genetic variations in olfactory pathway genes were associated with obesity, while smoking modified this effect.
	Case-control		Controls: 307 (39.1%; 51.2 ± 14.6; ≥ 18 kg/m^2^ and <24 kg/m^2^)	WC ≥90/≥85cm M/F		29 SNP's from 7 olfactory pathway related genes/receptors (*OR2AK2, OR2L8, OR4D1, OR52K1, OR52K2*, CALML3 and CLCA2)			
**Wang et al. (46)**	China	Explore the effects of rural-to-urban living environment transformation on MC4R gene polymorphisms and obesity	Cases: 322 (66.8%; ≥ 28 kg/m^2^	BMI ≥ 28 kg/m^2^	Differences in rural-to-urban living environments (education, income, smoking and drinking status and PA)	Candidate genes	The rs17782313 C allele and rs12970134 A allele were significantly related to obesity in Yi people.	Yi migrants had a greater obesity risk (OR = 2.59 [CI = 1.70, 3.95]) than Yi farmers. rs17782313 (AP = 0.65, [CI = 0.22,1.09]) and rs12970134 (AP = 0.59 [CI = 0.02, 1.17]) increased obesity risk in Yi migrants	The interaction between both *MC4R* SNPs and obesity risk was modified by the urban living environment
	Case-control		Controls: 643 (66.7%; < 24 kg/m^2^)			*MC4R* rs17782313 and rs12970134			
**Al-Jawadi et al. (29)**	Indonesia	Investigate association of *FTO* rs1421085 with BMI and macronutrient and fatty acid intake.	Cases: 35 (57.1%; 33 [27.5 – 39]; 31.86 [28.10 – 35.39])	BMI ≥ 25 kg/m^2^	Macronutrient (carbohydrate, protein, fat) and fatty acid (PUFA, MUFA, SFA) intake.	Candidate gene	Individuals with the CC genotype had a significantly higher BMI (β=12.58 [CI = 5.15, 20.01]), indicating a recessive trait	MUFA (β= 1.14 [CI = 0.02, 2.26]) and SFA (β=2.06 [CI = 0.29, 3.83]) were both positively associated with TC/CC genotypes compared to TT.	*FTO* rs1421085 CC and TC genotypes are positively associated with MUFA, SFA and increased BMI.
	Case-control		Controls: 36 (86.1%; 31 [27.5 – 34.6]; 20.86 [19.48 – 21.39])			*FTO* rs1421085			
**Daya et al. (31)**	Indonesia	Assess the interaction between *FTO* rs9939609, obesity and dietary fat intake.	Cases: 40 (85.0%; median (range) 31 (21-53) ; ≥25 kg/m^2^)	BMI ≥ 25 kg/m^2^	Daily total dietary fat intake	Candidate genes	The AT/AA genotypes were at higher risk of obesity (OR = 3.72 [CI = 1.19, 11.64]) and dietary fat intake (OR = 5.98 [CI = 1.22, 29.22]) compared to TT genotypes.	Obese AT/TT genotype individuals were significantly more likely to have high dietary fat intake than low fat intake compared to TT genotypes (OR = 1.40 [CI = 1.07-1.84])	*FTO* rs9939609 AT/TT genotypes are associated with increased risk of obesity and tendency towards high fat foods
	Case-control		Controls 40 (57.9%; median (range) 33 (19-52); <23 kg/m^2^)			*FTO* rs9939609			
**Rana et al. (37)**	Pakistan	Examine the effects of	Cases: 290 (45.2%; 30.7 ± 9.0; ≥ 25 kg/m^2^)	BMI ≥ 25 kg/m^2^	Random eating patterns (REP), tendency toward fat-dense food (TFDF), sleep duration, sleep–wake cycle (SWC), shift work (SW), and PA levels	Candidate genes	Only *TMEM18* rs7561317 was significantly associated with anthropometric traits such as increased BMI (*P*=0.045) and WC (*P*=0.045)	rs17782313, rs1421085, rs7561317, and rs2815752 genetic variants were shown to interact with REP, TFDF, irregular SWC, and low PA to increase obesity-related anthropometric indices	Genetic factors are the primary determinant of obesity susceptibility, however behavioural traits are shown to significantly modify this interaction.
	Case-control	gene–gene and gene–behaviour/lifestyle interactions on the risk of obesity in a Pakistani population	Controls: 288 (43.8%; 28.4 ± 8.4; < 25 kg/m^2^)			*MC4R* rs17782313, *BDNF* rs6265, *FTO* rs1421085, *TMEM18* rs7561317, and *NEGR1* rs2815752			
**Isgin-Atici et al. (33)**	Turkey	Assess the role of FTO rs9939609 and rs10163409 and their interaction with dietary intake and PA on obesity outcomes	Cases: 200 (46%; 36.37 ± 7; 29.04 ± 3.38)	BMI ≥ 25 kg/m^2^	Dietary intake (carbohydrate, protein, fibre, fat) and PA levels (sedentary vs active)	Candidate genes	*FTO* rs9939609 and the GRS was significantly associated with higher BMI and fat mass index (*P*=0.002 and *P*=0.003 respectively)	Higher protein intake was significantly associated with increased WC for *FTO* SNP rs10163409 carriers (*P*=0.044)	The impact of *FTO* SNPs on obesity may be moderated by dietary protein intake and PA.
	Case-control		Controls: 200 (50%; 33.29 ± 6.83; 22.56 ± 1.78)			*FTO* rs9939609 and rs10163409, and combined GRS			
**Gong et al. (40)**	China	Explore the effect of the gene–environment interaction on BMI, WC, and obesity among Chinese adults born in the 1960's.	2216 (60.3%; 49.7 [48.7 – 51.3]; 24.0 [21.9–26.4])	BMI ≥28.0 kg/m^2^ OR	PA (leisure-time PA, housework, transportation mode), SES (economic and education level), LTSB and dietary energy intake	Candidate genes	The effect of *MC4R* rs12970134 on BMI, and *TRHR* rs7832552 and *BCL2* rs12454712 on WC was attenuated by PA	LTSB, higher SES and higher energy intake increased the impact of SNPs on BMI and WC	High PA, low SES, reduced LTSB and low dietary intake have a negative association between SNP genetic susceptibility and obesity.
	Cross-sectional			WC ≥90/≥85cm M/F		12 obesity-related SNPs			
**Xue et al. (38)**	China	Examine the associations between types of PA and sedentary behaviours on anthropometric measures and their interaction with obesity genetic susceptibility	3976 (54.9%; median age 44.8)	BMI ≥ 25 kg/m^2^	PA (moderate to vigorous) and sedentary behaviours (time spent watching television, computer/ phone screen use)	Weighted GRS of 9 SNPs	For individuals with high GRS, there was a significant negative association between moderate to vigorous PA and WC and %BF	For those with high GRS, time spent watching TV was associated with high BMI: for every 1hr of TV watching, BMI, WC and %BF increased by 0.2kg/m^2^, 0.9cm and 0.3% respectively (*P*<0.02)	In those with high genetic risk of obesity, moderate to vigorous PA may reduce the risk of obesity, whilst prolonged TV watching may accentuate.
	Cross-sectional		Overall BMI distribution unavailable.						
**Alsulami et al. (30)**	Ghana	Investigate the effect of GRS on obesity-related traits and any modifying effects by dietary intake and PA levels.	302 (58.3%; 38.17 ± 9.64; 26.6 ± 5.0)	BMI ≥ 25 kg/m^2^	Dietary protein, fibre and fat intake (SFA, MUFA, PUFA) and PA levels.	Unweighted GRS of 4 SNPs (*TCF7L2* rs12255372, rs7903146, *MC4R* rs17782313, *FTO* rs9939609)	No association between GRS and any obesity-related traits.	Significant interaction between GRS ≥ 3 risk alleles and high total fat intake ( >47g/day) on WC (β= 71.28 ± 23.68). SFA, MUFA, PUFA intake on WC significant but not PA.	Higher consumption of total fat, SFA, MUFA and PUFA can increase genetic susceptibility to obesity.
	Cross-sectional								
**Moore et al. (39)**	India	Evaluate the association between 16 obesity-related SNPs and BMI and WC and moderating effects of PA levels.	New Delhi: 511 (54%; 47.1 ± 9.9; 19.6% obese)	BMI ≥30 kg/m^2^ OR	PA level (<81, 81-143, 144-211, >212 MET-h/wk)	Candidate genes	*FTO* rs3751812 T-allele was significantly associated with increased WC (1.58cm [CI = 0.60, 2.56] waist size increase per allele)	In participants with low PA (<81 MET–h/wk), T-allele was associated with increased WC (+2.68 cm [CI = 1.24, 4.12]) while high PA (>212 MET–h/wk had no association.	*FTO* rs3751812 genetic susceptibility to obesity could be attenuated by high levels of PA
	Cross-sectional		Trivandrum: 618 (48.7 ± 9.2; 17.5% obese)	WC ≥90/≥80cm M/F		16 SNPs in or near *FTO, MC4R, G6PC2, GCKR, TCF7L2*, and *SLC30A8* genes			
**Wuni et al. (32)**	India	Investigate the effect of a GRS on obesity-related traits and any moderating effects of dietary intake	497 (54.7%; 44 ± 10; 24.6 ± 4.5)	BMI ≥ 25 kg/m^2^	Dietary protein, carbohydrate, fibre and fat intake (SFA, MUFA, PUFA) and total energy intake	Unweighted GRS of 3 SNPs (*LPL* rs327, rs3200218 and *CETP* rs4783961)	No association between the GRS and obesity related traits (HDL, LDL, TG, total cholesterol, SBP, DBP, BMI, WC, WHR)	In individuals with high GRS (>2 risk alleles) high SFA intake was associated with increased WC compared to low SFA intake (β = 0.02, *P*=0.02)	SFA intake may modify the genetic risk of lipid-pathway genes SNPs on obesity
	Cross-sectional								
**Mahmoudi-Nezhad et al. (35)**	Iran	Assess the interaction of *CARTPT* rs2239670 genotypes and dietary indices on anthropometric measures in obese individuals.	287 (51.1%; M: 38.44 ± 6.86, F: 37.81 ± 8.25; M: 33.90 ± 3.41, F: 35.61 ± 4.31)	BMI 30–40 kg/m^2^	Dietary indices (healthy eating index (HEI) and diet quality index – international (DQI-I)	Candidate genes	Significant *CARTPT*–HEI interactions for BMR, serum glucose and HDL concentrations. HEI could not modify adverse effects of *CARTPT* rs2239670 AA genotype	*CARTPT*–DQI-I interactions were more pronounced compared to *CARTPT*–HEI interactions for fat mass (*P*=0.02), WC (*P*=<0.001), and BMR (*P*=<0.001).	*CARTPT* rs2239670 genotype was significantly associated with HEI and particularly DQI-I scores for BMR, WC and FM
	Cross-sectional					*CARTPT* rs2239670			
**Rahati et al. (36)**	Iran	Investigate the impact of behavioural characteristics on the association between near *CLOCK* rs1801260 and obesity	403 (36.5 ± 8.7; 30.2 ± 3.1)	Overweight + obese: BMI 25–40 kg/m^2^	Energy and macronutrient intake, circadian rhythm, sleep duration and food timing	Candidate genes	Significant difference between *CLOCK* rs1801260 genotype study groups for energy and macronutrient intake, food timing, sleep and PA	Eating lunch after 3pm significantly increased obesity susceptibility (OR= 2.95 [CI = 1.77, 4.90]) in CT + CC *CLOCK* rs1801260 genotype carriers	*CLOCK* C allele carries are more likely to experience higher energy intake, reduced sleep and later meal timings, and are more genetically susceptible to obesity if eating lunch after 3pm.
	Cross-sectional		No information on gender distribution	No individual classification		*CLOCK* 3111 T / C			
**Illangasekera et al. (45)**	Sri Lanka	Investigate the role of *FTO* and near *MC4R* SNPs on obesity measures and the moderating effects of urban and rural living	528 (60.9%; M 47.2 ± 11.6, F 47.5 ± 11.5)	BMI ≥ 25 kg/m^2^ and ≥ 27.5 kg/m^2^	Urban vs rural living	Candidate genes	FTO rs9939609 (AA +TT) carriers and near MC4R rs17782313 (CC+TT) had a significantly higher BMI, and were associated with categorial obesity.	FTO rs9939609 (AA +AT) was associated with significantly greater mean BMI in urban populations vs rural (M(SE) = 1.20(0.53), *P* = 0.02)	*FTO* and *MC4R* SNPs are associated with obesity, and urban living may accentuate the obesogenic effect of the *FTO* SNP.
	Cross-sectional		No information on overall BMI distribution			*FTO* rs9939609 and near *MC4R* rs17782313			

BCL2; B-cell lymphoma-2, BDNF; brain-derived neurotrophic factor, BMI; body mass index, BMR; basal metabolic rate, CALML3; calmodulin like 3, CARTPT; cocaine and amphetamine-regulated transcript prepropeptide, CETP; cholesteryl ester transfer protein, CI; confidence interval, CLCA2; chloride channel accessory 2, CLOCK; circadian locomotor output cycles kaput, DBP; diastolic blood pressure, DQI-I; diet quality index – international, F; female, FLJ33534; putative uncharacterized protein, FM; fat mass, FTO; fat mass and obesity-associated, GCKR; glucokinase regulatory protein, GRS; genetic risk score, G6PC2; glucose-6-phosphatase catalytic 2, HDL; high density lipoprotein, HEI; healthy eating index, LDL; low density lipoprotein, LTSB; leisure time sedentary behaviour, M; male, MC4R; melanocortin 4 receptor, MET; metabolic equivalent of task, MUFA; monounsaturated fatty acids, NEGR1; neuronal growth regulator 1, OR; odds ratio, OR2AK2; olfactory receptor family 2 subfamily AK member 2, OR2L8; olfactory receptor family 2 subfamily L member 8, OR4D1; olfactory receptor family 4 subfamily D member 1, OR52K1; olfactory receptor family 52 subfamily K member 1, OR52K2; olfactory receptor family 52 subfamily K member 2, PA; physical activity, PUFA; polyunsaturated fatty acids, REP; random eating patterns, SBP; systolic blood pressure, SES; socioeconomic status, SFA; saturated fatty acid, SLC30A8; solute carrier family 30 member 8, SNP; single nucleotide polymorphism, SW; shift work, SWC; sleep–wake cycle, TCF7L2; transcription factor 7-like 2, TFDF; tendency toward fat-dense food, TMEM18; transmembrane protein 18, TRHR; thyrotropin-releasing hormone receptor, UCP2; uncoupling protein 2, WC; waist circumference, WHR; waist hip ratio, %BF; percentage body fat.

Data are expressed as mean (standard deviation) or count (%) unless otherwise specified.

**Table 3:** Summary of included gwas investigating gene-environment interactions on obesity.

Authors (year)	Study country/ design	Study objectives	Sample size (female %; age distribution; bmi distribution)	Obesity definition	Lifestyle/ environmental factors	Genetic approach	Primary results	Secondary results	Conclusion
**Ahmad et al. (43)**	Pakistan Cross-sectional	Use genome wide approaches to conduct gene-lifestyle interaction analyses for smoking and PA in relation to obesity.	GWAS: 14,131 (17.7%; 53.8 ± 9.6; 25.7 ± 4.2) Interaction analysis: 8,193, age: 30-80 No further distribution information	–	Tobacco smoking (never, ever, current) and PA (light, moderate, heavy)	vGWAS *FLJ33544* rs140133294	Lead variant identified (*FLJ33534*; rs140133294); with strong association on BMI phenotypic variance (*P*=3.1 × 10^-8^)	Association of rs140133294 (*FLJ33534*) with BMI stratified by smoking status: never smokers (β=0.90 ± 0.36); current smokers (β=−1.51 ± 0.52). No significant association for PA.	Single *FLJ33534* locus significantly modifies the relationship of smoking and BMI

GWAS; genome-wide association study, vGWAS; variance heterogeneity genome-wide association study, PA; physical activity Data are expressed as mean (standard deviation) or count (%) unless otherwise specified.

#### Gene-environment exposures

Four articles investigated various environmental or lifestyle factors and their genetic interactions on the risk of obesity. Five studies assessed dietary interactions only, including nutritional components and dietary patterns; three studies investigated the effects of physical activity and sedentary behaviours; three studies looked at smoking and drinking statuses; two studies evaluated sleep patterns, and two studies assessed urban-rural differences and effects of within-country migration. Concerning genetic exposures, there was only one GWAS included in this review. Four studies assessed genetic risk through a genetic risk score (GRS), with the number of included SNPs ranging from 2 to 9. Only two studies assigned weights using an external independent study or genome-wide meta-analyses, whilst the other three used an unweighted approach. Thirteen studies used a candidate gene approach to investigate 62 different SNPs, with risk alleles in genetic variants of the *FTO* and melanocortin 4 receptor (*MC4R*) gene most commonly studied. For only two studies, SNPs selection was based on recent GWAS conducted in the same ancestral population as the study participants, while the rest either relied on GWAS of European ancestry or failed to justify.

#### Obesity outcomes

Anthropometric indices for obesity outcomes included (change in) weight, BMI, waist circumference (WC), hip circumference (HC), waist-hip ratio (WHR), waist-to-height ratio (WHtR), fat mass index (FMI), fat-free mass index (FFMI), and percentage body fat (%BF). Population measures were also examined, including odds of being overweight. For all included studies, obesity outcomes were objectively measured by a healthcare provider or study investigators using validated and compatible devices. Definitions of general obesity ranged across the studies, countries, and populations from BMI ≥ 25 kg/m^2^ to BMI ≥ 30 kg/m^2^.

### Association studies

#### Diet and food timing

Studies investigating gene-diet interactions on obesity focus on macronutrient intake, including total fat, protein and carbohydrates, and fatty acid intake, including saturated (SAFA), monounsaturated (MUFA), and polyunsaturated (PUFA) fatty acids were most prevalent. Within these studies, Al-Jawadi et al. (n = 71), Alsulami et al. (n = 302), Daya et al. (n = 80) and Wuni et al. (n = 497) all reported significant associations between higher total fat intake and obesity traits, for those carrying risk alleles of obesity-related gene variants, or for those with a high GRS in Indonesian, Ghanaian and Indian populations (29– 32). High SFA was also found to interact positively with WC in those with increased genetic susceptibility to obesity in the cross-sectional studies by Alsulami et al. and Wuni et al. Individuals with high GRS (≥ 2 risk alleles) and high SFA intake (>14 g/day) had a significantly higher WC (*P*_interaction_ = 0.02) compared to those with low SFA intake after adjustment for age and sex in the Ghanaian study. In contrast, in the Indian population, those with lower SFA intake (≤23.2 g/day) had a significantly smaller WC (β= -0.01cm, *P*=0.03) (*P*_interaction_= 0.006) after adjustment for age, sex and 6 other potential confounders (30, 32). However, some inconsistencies were also reported on the modifying effects of dietary fat intake on genetic susceptibility and obesity risk. In the case-control study by Isgin-Atici et al. (n = 400), the same *FTO* SNP rs9939609 variant as the Indonesian case-control study by Daya et al. was investigated individually and as part of a GRS with one other *FTO* gene variant. In contrast, no statistically significant association was found between dietary fat intake and obesity traits in Turkish populations (33). Findings concerning dietary protein intake and its interaction with *FTO* gene variants on obesity measures also conflicted in Turkish and Indonesian populations. In the study by Isgin-Atici et al., carriers of FTO risk alleles showed a significant interaction with protein intake on increased WC (*P*_interaction_ = 0.044) after adjustment for age, sex hypertension and CVD.

Concerning dietary patterns, two studies examining eating patterns in two separate Iranian populations reported modifying effects in individuals with either high GRS or those carrying risk alleles of the Cocaine and Amphetamine-Regulated Transcript Prepropeptide (*CARTPT*) gene and their association with obesity-related anthropometric measures. In a cohort study, Hosseini-Esfahani et al. (n = 4,292) showed that higher Western dietary pattern scores (namely high intakes of processed foods and drinks, sugar, red meat, and high-fat dairy) were associated with increased BMI in subjects with high GRS compared to those with low GRS over time (*P*_interaction_= 0.01) after multivariable adjustment for age, sex and 5 other confounders (34). In a cross-sectional study, Mahmoudi-Nezhad et al. (n = 287) used the Diet Quality Index—International (DQI-I), an indicator of nutritional variety, moderation, and adequacy to show that in individuals with high-scoring quality diets, *CARPT*-DQI-I interactions significantly reduced BMI (*P*_interaction_ < 0.001) following adjustment for age and sex (35). In both studies, however, analyses assessing healthy eating patterns rich in fruits, vegetables, fish and whole grains, quantified by the Healthy Eating Index, showed no significant modifying effects by genotypic groups or GRS for obesity traits.

Significant interactions between food timing and genetic variants on obesity were also demonstrated in both Iranian and Pakistani populations. Rahati et al. (n = 403) reported in a cross-sectional study that for carriers of Circadian Locomotor Output Cycles Kaput (*CLOCK*) gene polymorphisms, delayed eating times for breakfast and lunch increased the odds of obesity by 2.95 (95% CI = 1.77, 4.90) and 1.53 (95% CI = 1.32, 1.89) respectively after adjustment for age, sex and 6 other confounders (36). Significant interactions between risk alleles in multiple genes, including *FTO, MC4R*, and transmembrane protein 18 (*TMEM18)* and random eating patterns were also found to increase BMI (*P*_interaction_=0.002, *P*_interaction_ = 0.008, *P*_interaction_=0.001 respectively) in the case-control study by Rana et al. (n = 578) focussing on a Pakistani population after age and sex adjustment (37).

#### Physical activity and sedentary behaviours

A total of 10 studies using either a candidate gene approach (n = 6) or GRS (n = 4) reported gene-physical activity (PA) interactions on obesity traits. PA or sedentary behaviour were defined *via* participant self-reporting in studies by Xue et al., Rana et al., Moore et al., Isgin-Atici et al., and Gong et al. (33, 37–40) while articles by Sun et al., Muhammed et al., Alsulami et al., and Ahmad et al., used investigator administered questionnaires (30, 41–43). The standardised international physical activity questionnaire was most used to assess PA levels across studies (n = 4), which assesses levels of PA relating to work and house-related work, transportation, and recreation, calculated and summarised as metabolic equivalent of task units per week (MET-min/wk).

Four candidate gene studies showed that interactions between 4 different gene variants and low levels of PA were significantly associated with obesity-related anthropometrics (33, 37, 39, 42). The study by Moore et al. (n = 1,129) used a cross-sectional design to show that in India, for participants with a low PA level of <81 MET-h/wk, the *FTO* s3751812 risk allele was significantly associated with an increased WC (β = 2.68; 95% CI = 1.24, 4.12) after controlling for age, sex, region, and religion (39). The same association for variants of the *FTO* candidate gene was also found in 2 other case-control studies, which focused on Turkish (n = 400) and Pakistani populations (n = 578) (33, 37). High levels of PA, defined as >212 MET-h/wk, did not produce any significant gene interaction effect on obesity (39). In conflict, there were no significant interactions between putative uncharacterized protein (*FLJ33534*), uncoupling protein 2 (*UCP2*), or olfactory pathway-related candidate genes and PA, or their interaction on obesity-related traits (41–43). The study by Alsulami et al., which used a GRS approach comprised partly of *FTO* gene variants, also failed to show any significant interaction between high GRS and PA on obesity (30) in a Ghanaian population. Two cross-sectional studies investigated sedentary behaviours and their potentially modifying effects in two Chinese people. Gong et al. (n = 2,216) and Xue et al. (n = 3,976) consistently showed that increased leisure time sedentary behaviours such as television watching positively accentuated the interaction between high GRS or SNPs and WC and BMI after multivariable adjustment (38, 40).

#### Tobacco smoking and alcohol consumption

Across the studies included in this review, very few investigated GxE assessing the effect of tobacco smoking (n = 3) or alcohol consumption (n = 2) on obesity. In these candidate gene studies, tobacco and alcohol exposures were assessed by a self-administered questionnaire by Wei et al., while Ahmad et al. and Sun et al. collected data using researcher administered validated questionnaires in the form of a structured interview (41, 43, 44). Both Ahmad et al. (n = 8,193) and Sun et al. (n = 608), using cross-sectional and case-control study designs, respectively, showed that for current smokers, the interaction between smoking status and obesity was modified by different gene variants (41, 43). In the current smokers from Pakistan, *FLJ33534* risk alleles showed a negative association with BMI (β=−1.51 ± 0.52, *P*=0.003) after adjustment for age, sex, and genetic ancestry (43). While the Chinese population showed smoking increased the risk of obesity for those with high olfactory receptor family 4 subfamily D member 1 (*OR4D1*) gene scores (OR = 2.67; 95% CI = 1.35, 5.30; *P=*0.005; *P_interaction_* = 0.041) but decreased the risk of obesity for those with high calmodulin-like 3 (*CALML3*) gene scores (OR = 0.25; 95% CI = 0.10, 0.62; *P*=0.003; *P_interaction_* = 0.026) after adjustment for age, sex, PA and alcohol consumption (41). However, a separate case-control study in China by Wei et al. (n = 1,836) showed no significant gene-smoking or gene-alcohol interaction on obesity risk for *MC4R* genotypes (44). Alcohol consumption was also consistently disproved to show any modifying effect on the relationship between gene variants and obesity by Sun et al. (41).

#### Sleeping patterns

Only two candidate gene studies performed gene-sleep interaction analyses on obesity traits, both of which estimated sleeping patterns and sleep duration using participant self-reporting *via* study-specific questionnaires. Both studies demonstrated unfavourable outcomes on obesity traits in response to the interaction between genetic variants and reduced sleeping times. In a Pakistani population (n = 578), Rana et al. applied a case-control study design to demonstrate a significant interaction between *TMEM18*, neuronal growth regulator 1 (*NEGR1*)*, FTO* and *MC4R* gene variants and irregular sleep-wake cycle, which was shown to augment BMI, WC, HC, WHR, WHtR and %BF, in carriers of risk alleles after controlling for age and sex (37). In the same study, inadequate sleep, defined as <7 hours/night, was also shown to interact with *FTO, TMEM18* and *NEGR1* gene variants to increase BMI and WC significantly. In a separate cross-sectional study by Rahati et al., the interaction between sleep duration (hours/week) and *CLOCK* rs1801260 genotypes were also assessed in an Iranian population (n = 403), where obese individuals with the CT and CC genotypes had a significant shorter sleeping time than TT genotype carriers after controlling for age, sex and 6 other variables (36).

#### Rural-urban differences

Two studies investigating the moderating effects of urban and rural living environments found a disparity in their effects on obesity and their interactions with *MC4R* candidate genes in Chinese and Sri Lankan populations. Information about sociodemographic information and lifestyle factors was collected through face-to-face interviews or participant self-report *via* standardised questionnaires by Wang et al. and Illangasekera et al., respectively. The *MCR4* rs17782313 CC and CT genotype were cross-sectionally associated with significantly higher BMI values in Sri Lankans (*P*= 0.03) (n = 528) compared to the TT genotype, a result which was replicated in the Chinese case-control study by Wang et al. (n = 965), which demonstrated significantly higher odds of obesity (OR = 3.01; 95% CI = 1.49, 6.05) for homozygous C allele carriers (45, 46). However, on the performance of stratified analysis by urban or rural residence and the interaction with the *MC4R* gene polymorphism on obesity, only the study by Wang et al. found a statistically significant heterogeneous association between the two living environments, with an attributable proportion of 0.65 (95% CI = 0.22, 1.17) after controlling for age, sex and 7 other potential confounders (46). In contrast, Illangasekera et al. showed the *MC4R* non-variant TT carriers of urban residence to record higher mean BMIs (45).

## Discussion

To our knowledge, this is the first review to provide an overview of current literature investigating the effects of gene-environment interactions on obesity traits in LMICs. Approximately two-thirds of the 18 studies (n=26,684) included in our review explored gene-diet or gene-physical activity interactions. In contrast, more limited numbers explored other emerging obesogenic environmental risk factors, such as urbanicity, irregular or insufficient sleep, and tobacco and alcohol use. Results from this study indicate there may be some consistent associations across LMICs for interactions between genetic variants and reduced sleeping times, urban living environments, low levels of PA, increased sedentary behaviour and delayed eating patterns on obesity outcomes. However, due to considerable heterogeneity between study outcome definitions, genetic polymorphisms, and environmental and lifestyle factors, in combination with the genetic heterogeneity across different ethnic groups in LMICs, genotype-phenotype cross-correlations should be interpreted with caution.

While results from nutrigenetic studies and studies investigating gene-physical activity interactions on obesity suggest there could be some consistent associations between eating patterns or low levels of PA and their genetic interactions on an increased risk of obesity, associations with specific gene variants were not replicated across studies. Across all included studies, statistically significant findings were reported for 12 individual SNPs, including *FLJ33544* rs140133294 (43), *FTO* rs1421085 (29, 37), rs9939609 (31, 45), rs10163409 (33), rs3751812 (39), *CARTPT* rs2239670 (35), *UCP2* rs659366 (42), *CLOCK* rs1801260 (36), *MC4R* rs17782313 (37, 45, 46), rs12970134 (40, 46), *TMEM18* rs7561317 and *NEGR1* rs2815752 (37). However, these associations were either only significant in single trials or were not replicated in response to the same environmental exposure. Trials that assessed interactions between the same *FTO* genetic variants and macronutrients, including total fat and protein intake on obesity, conflicted across different LMICs and populations (29, 31, 33).

Explanations for these inconsistent results could be accounted for in the substantial heterogeneity in exposure and outcome data collection methods, alongside wide-ranging obesity definitions in the included studies. Obesity and overweight were often not differentiated, and using BMI values only as a screening tool for obesity was expected, with cut-offs varying from ≥ 25 kg/m^2^ to ≥ 30 kg/m^2^. Further heterogeneity can be evidenced by the additional incorporation of WC measurements into obesity definitions by some studies to measure central obesity. Regional and ethnic differences in anthropometry and adiposity prevent the use of standardised global obesity definitions, as evidenced by WHO guidelines, which define obesity as 2.5 kg/m*^2^* lower in Asian populations compared to the global standard (2, 47). However, this review has demonstrated disparity in the use of obesity definitions even amongst populations from the same developing countries, signalling the need for a more consistent application of recommended definitions. Assessment and definitions of exposures were also conflicting across studies, and at high risk of recall or reporting bias due to incomplete or ambiguous recording of methods and use of short self-developed questionnaires and surveys for patient self-report. Confounding was controlled reasonably well across studies, with age- and sex-adjusted for by all studies as a minimum.

There is a high prevalence of statistically ‘significant’ GxE on obesity on single genetic variants or environmental exposures without replication. However, even with replication, many of the study designs shown in this review are susceptible to reverse causation, highlighting the need for more well-controlled long-term prospective longitudinal research looking at GxE on obesity. Using *P*-values without effect sizes or confidence intervals to report associations was also prevalent across studies (32–35, 42), alongside small sample sizes and erroneous underpowered statistical analyses, resulting in concerns for selective reporting and publication bias.

From what is demonstrated in this review, emerging primary research from LMICs investigating GxE primarily employs a hypothesis-based approach, using pre-specified genes of interest identified in GWAS studies from European populations. The inconsistencies and lack of replication across study findings could be partly attributed to the use of genetic variants identified from GWAS conducted in developed regions with ancestrally homogenous populations. It is, therefore, likely that the inconsistency in study findings results from a lack of generalisability of the GWAS-identified candidate genes from developed nations due to the varied genetic architecture found in the diverse ethnic populations across LMICs (48). In addition, of the studies using a candidate gene approach, approximately half failed to correct for multiple testing when using multiple regression analysis to investigate several SNPs across different genetic loci (49). Of those which did control for Type I error, sample sizes were very small and consequentially underpowered to detect GxE reliably.

Only five of the studies included in this review used a GRS (30, 32–34, 38), which, while eliminating the loss of statistical power attributable to correction for multiple testing, studies were still underpowered owing to insufficient sample sizes of 400, 497 and 302 for studies by Isgin-Atici et al., Wuni et al. and Alsulami et al. respectively (30, 32, 33, 50). Moreover, only two studies by Xue et al. and Hosseini-Esfahani et al. assigned a weighted method using external weights from an independent study of the same ancestral population (38) or a multi-ethnic GWAS meta-analysis, respectively (34). The absence of suitable external weights for the studies using unweighted GRS in Turkish, Indian and Ghanaian populations further demonstrates the lack of global diversity in the existing genetic research (30, 32, 33). As an aggregation of multiple genetic variants, weighted GRS using meta-analysed external weights is considered the gold standard for this genetic approach and is a powerful and bourgeoning tool for identifying GxE (51). New statistical techniques which rely on internal information on effect size distributions could improve the accuracy of research in developing countries using GRS analyses where external genetic information is unavailable (52, 53).

Applying more GWAS approaches in LMIC research would eliminate reliance on the strength of *a priori* evidence and any previous associations drawn from homogenous populations of European descent, producing quality genetic associations for future gene-interaction studies (54). Only one paper eligible for inclusion in this study used a GWAS approach in a Pakistani population to identify the *FLJ33534* obesogenic locus. This paper demonstrated a robust and high-quality significant interaction between the identified genetic variant, smoking and a moderating effect on obesity (43). Transethnic GWAS studies to estimate improved GRS could provide greater predictive power for future GxE studies in developing countries, and their inclusion in future research has been called for in previous reviews (18, 55). However, with a recommended genome-wide significance threshold of *P* = 5x10^-8^, GWAS require huge sample sizes to reach an adequate statistical power (56). This presents a significant challenge in LMICs, where resources are often limited, and participant recruitment can be challenging due to low engagement levels and distrust of the scientific community (48). Expansion of genetic studies in diverse populations is essential, and while an increase in research capacity from LMICs could eliminate the Eurocentric biases surrounding GWAS and GxE interactions, a more equitable and open sharing of technologies, statistical advancements and GWAS summary statistics in diverse populations is needed to improve the quality of future research and eliminate health disparities (57).

Recent research from HICs has shown that environmental factors can influence an individual’s genetic susceptibility to obesity. For example, a recent study of 331,282 participants in the UK Biobank found that metabolic equivalent task (MET) score, pack-years of smoking, and alcohol intake frequency significantly interact with genetic factors related to obesity for BMI (58). Findings from GxE research in HICs cannot be assumed to be the same in LMICs. This is because people living in LMICs have different ethnic backgrounds with different genetic information and live in different environments with different lifestyles.

### Study strengths and limitations

The strength of this systematic review is that it is the first to report on GxE on obesity traits in LMICs through a broad and exhaustive literature search, using rigorous and predetermined inclusion and exclusion criteria. Evidence of this topic as a fast-developing and emerging field of research in LMIC countries can be substantiated by the number of included primary research conducted or published in the last two years. However, several limitations associated with this study should be highlighted. Despite nearly all included studies reporting significant findings for GxE on obesity, the limited homogeneity among studied SNPs and outcome definitions severely restricted the synthesis and interpretation of results. In addition, no meta-analysis or quantitative data synthesis could be performed, owing to the considerable heterogeneity and sources of error surrounding the included primary research. Imprecise and diverse measurements of exposures and outcomes, alongside small sample sizes and underpowered or improper interaction analyses, which could have yielded false positive or false negative results, mitigate any value in a meta-analytic summary of effect sizes of GxE. It should also be noted that although the search strategy and study eligibility criteria did not exclude studies based on language, only English search terms were used in database searches. In addition, no non-English databases were included in the search strategy. This could explain the lack of any representation of research from developing countries from Central and South America and the limited representation of studies from developing African countries eligible for inclusion in this review, with only three irrelevant non-English studies identified in the initial database search. Finally, we didn’t assess the quality of the reviewed studies since scoping reviews offer a summary of the evidence available rather than synthesising results for clinical implementations as systematic reviews.

## Conclusion

This review has examined and discussed population-based studies from LMICs investigating GxE and their effect on obesity, in addition to synthesising the achievements and pitfalls in the currently available primary research. Individual results have shown smoking status to modify the interaction between *FLJ33544* and olfactory pathway genetic loci and obesity traits. At the same time, urban living environments were demonstrated to interact with *MC4R* gene polymorphisms to increase obesity traits. However, the ability to draw concrete conclusions is limited due to concerns over study quality and a high potential for biases. The considerable heterogeneity exhibited across the investigated genetic variants, exposure and outcome measures, statistical analyses, and data reporting has highlighted a need for updated standardised protocols bespoke to LMICs, and advanced statistical techniques and data availability to improve the quality and comparability of future studies.

## Author contributions

FA is responsible for the study concept and design. SLP performed the literature review and wrote the manuscript. All authors revised/edited the manuscript for intellectual content. All authors contributed to the article and approved the submitted version.
