# Supplementary material for: Gene-environment interactions and the effect on obesity risk in low and middle-income countries: a scoping review
Source: Front Endocrinol (Lausanne). 2023 Aug 18;14:1230445. doi: 10.3389/fendo.2023.1230445 (PMC10474324; doi:10.3389/fendo.2023.1230445)
Supplement: Supplementary file 1 [file Table_1.docx]

**Supplementary Tables**

**SUPPLEMENTARY TABLE 1 PubMed Medline Search Strategy**

| Database: PubMed MEDLINER <up to 24^th^ October 2022> | | | Results |
| --- | --- | --- | --- |
| Population | 1 | “Developing Countries”[Mesh] OR “developing countr*”[tiab] OR “under developed countr*”[tiab] OR lmic*[tiab] OR “less developed”[tiab] OR “low income”[tiab] OR “lower income”[tiab] OR “low and middle income”[tiab] OR “low middle income”[tiab] OR “resource poor”[tiab] OR “resource constrained”[tiab] OR “low resource”[tiab] OR “limited resource*”[tiab] OR “resource limited”[tiab] | 239,258 |
|  | 2 | “Africa South of the Sahara”[Mesh] OR “Central America”[Mesh] OR “South America”[Mesh] OR “Latin America”[Mesh] OR “Caribbean Region”[Mesh] OR “Mexico”[Mesh] OR “Asia”[Mesh] OR “China”[Mesh] OR “North Korea”[Title/Abstract] OR “Mongolia”[Mesh] | 1,516,222 |
|  | 3 | #1 OR #2 | 1,658,799 |
| Determinant | 4 | "Gene-Environment Interaction"[Mesh] OR “Gene X environment”[Title/Abstract] OR “gene-by-environment” [Title/Abstract] OR “g x e” [Title/Abstract] OR GxE [Title/Abstract] “gene-environment” [Title/Abstract] OR “gene environment”[Title/Abstract] OR “gene-lifestyle interaction” [Title/Abstract] OR “gene lifestyle interaction” [Title/Abstract] | 8,807 |
|  | 5 | "Genome-Wide Association Study"[Mesh] OR GWA[Title/Abstract] OR “Genome-Wide Association”[Title/Abstract] OR “Polygenic score” [Title/Abstract] OR “polygenic risk score”[Title/Abstract] OR “PGS” [Title/Abstract] OR “PRS” [Title/Abstract] OR "Polymorphism, Single Nucleotide"[Mesh] OR SNP [Title/Abstract] OR “single nucleotide polymorphisms”[Title/Abstract] OR “gene variant”[Title/Abstract] OR “genotype”[Title/Abstract] OR “genetics” [Title/Abstract] OR “obesity-associated gene*” [Title/Abstract] OR “epigenetic”[Title/Abstract] OR “methylation”[Title/Abstract] | 683,962 |
|  | 6 | Environment [Title/Abstract] OR obesogenic[Title/Abstract] OR lifestyle[Title/Abstract] OR “physical activity”[Title/Abstract] OR “physical inactivity” [Title/Abstract] OR exercise*[Title/Abstract] OR diet*[Title/Abstract] OR smok* [Title/Abstract] OR alcohol [Title/Abstract] OR sleep* [Title/Abstract] | 2,408,124 |
|  | 7 | #5 AND #6 | 69,164 |
|  | 8 | #7 OR #4 | 73,166 |
| Outcome | 9 | “Obesity” [MeSH] OR Obesit*[Title/Abstract] OR “abdominal fat” [MeSH] OR “body weight” [Title/Abstract] OR “overweight” [Title/Abstract] OR adiposity[Title/Abstract] OR BMI[Title/Abstract] OR “body mass index” [Title/Abstract] OR weight[Title/Abstract] OR “waist circumference” [Title/Abstract] | 1,381,403 |
|  | 10 | #3 AND #8 AND #9 | 1,325 |
| Exclusion | 11 | “Adolescent”[Mesh] OR “child”[Mesh] OR adolescent*[Title/Abstract] OR child*[Title/Abstract] OR “infant*”[Title/Abstract] OR “teen*”[Title/Abstract] OR “pediatr*” [Title/Abstract] OR “paediatr*”[Title/Abstract] OR “birth” [Title/Abstract] | 4,343,961 |
|  | 12 | “Randomized Controlled Trial”[Publication Type] OR “controlled clinical trial”[Publication Type] OR randomized[Title/Abstract] OR randomised[Title/Abstract] OR placebo[Title/Abstract] | 1,109,309 |
|  | 13 | "animals"[mesh] NOT "humans"[mesh] | 5,065,614 |
|  | 14 | "Neoplasms"[Mesh] OR "Hypertension"[Mesh] | 4,059,674 |
|  | 15 | #11 OR #12 OR #13 | 13,323,044 |
|  | 16 | #10 NOT #14 | 744 |

**SUPPLEMENTARY TABLE 2 EMBASE Search Strategy**

| Database: EMBASE <up to 24^th^ October 2022> | | | Results |
| --- | --- | --- | --- |
| Population | 1 | (“Developing countr*” or “under developed countr*” or lmic* or “less developed” or “low income” or “lower income” or “low and middle income” or “low middle income” or “resource poor” or “resource constrained” or “low resource” or “limited resource?” or “resource limited”):ti,ab | 219,543 |
|  | 2 | (“Africa South of the Sahara” or “Central America” or “South America” or “Latin America” or “Caribbean” or “Mexico” or “Asia” or  “China” or “North Korea” or “Mongolia”):ti,ab | 512,579 |
|  | 3 | #1 OR #2 | 715,200 |
| Determinant | 4 | (“Gene-environment interaction" or “Gene X environment” or “gene-by-environment” or “g x e” or gxe or “gene-environment” or “gene environment” or “gene-lifestyle interaction” or “gene lifestyle interaction”):ti,ab | 11,446 |
|  | 5 | ("Genome-wide association" or GWA or “polygenic score” or “polygenic risk score” or PGS or PRS or “single nucleotide polymorphism?” or SNP or “gene variant” or genotype or genetics OR “obesity-associated gene?” or genotype or epigenetic? or methylation):ti,ab | 724,373 |
|  | 6 | (Environment or obesogenic or lifestyle or “physical activity” or “physical inactivity” or exercise? or exercising or diet? or dietary or smoking or smoke or alcohol or sleep?):ti,ab | 2,177,846 |
|  | 7 | #5 AND #6 | 63,888 |
|  | 8 | #4 OR #7 | 69,946 |
| Outcome | 9 | (obesity or “abdominal fat” or “visceral fat” or “body weight” or overweight or adiposity or BMI or “body mass index” or weight or “waist circumference”):ti,ab,kw | 1,915,847 |
|  | 10 | #3 AND #8 AND #9 | 496 |
| Exclusion | 11 | (Adolescent or child or children or infant or infancy or teen or teenager or pediatric or paediatric or birth):ti,ab | 2,764,505 |
|  | 12 | ‘Animal model’/exp or ‘animal experiment’/exp or (mice or mouse or pig or primate or fish or rat or rats or rabbit or rabbits or monkey or monkeys or cat or cats or dog or dogs). ti,ab,kw. | 5,447,938 |
|  | 13 | 'clinical trial'/de OR 'randomized controlled trial'/de | 1,483,375 |
|  | 14 | 11 OR 12 OR 13 | 9,375,128 |
|  | 15 | 10 NOT 14 | 133 |

**SUPPLEMENTARY TABLE 3 Scopus Search Strategy**

| Database: Scopus <up to 24^th^ October 2022> | | | Results |
| --- | --- | --- | --- |
| Population | 1 | TITLE-ABS-KEY (“Developing countr*” OR “under developed countr*” OR lmic* OR “low* income” OR “low middle income” or “resource poor” OR “low resource” OR “limited resource*” OR Africa OR “Central America” OR “South America” OR “Latin America” OR Caribbean OR Mexico OR Asia OR China OR “North Korea” OR Mongolia | 2,769,140 |
| Determinant | 2 | TITLE-ABS-KEY (“Gene-environment” OR “Gene X environment” OR “gene-by-environment” OR gxe OR “gene lifestyle interaction”) | 17,352 |
|  | 3 | TITLE-ABS-KEY ("Genome-wide association" OR GWA OR polygenic OR PGS OR PRS OR polymorphism* OR SNP OR “gene variant” OR genotype OR genetics OR “obesity-associated gene?” OR epigenetic*) | 3,185,743 |
|  | 4 | TITLE-ABS-KEY (Environment OR obesogenic OR lifestyle OR “physical activity” OR “physical inactivity” OR exercise* OR diet* OR smok* OR alcohol OR sleep*) | 7,366,799 |
|  | 5 | #3 AND #4 | 335,069 |
|  | 6 | #2 OR #5 | 337,526 |
| Outcome | 7 | TITLE-ABS-KEY (Obesity OR overweight OR adiposity OR BMI OR “waist circumference”) | 710,542 |
|  | 8 | #1 AND #6 AND #7 | 1,474 |
| Exclusion | 9 | TITLE-ABS-KEY (Adolescent OR child* OR infan* OR teen* OR pediatric OR paediatric OR birth OR “animal model” OR “animal experiment” OR mice OR mouse OR pig* OR primate OR fish OR rat* OR rabbit* OR monkey* OR cat* OR dog* OR “clinical trial” OR “randomized controlled trial”) | 28,611,345 |
|  | 10 | #8 AND NOT #9 | 499 |

**REFERENCES**

1. Herzog R, Álvarez-Pasquin MJ, Díaz C, Del Barrio JL, Estrada JM, Gil Á. Are healthcare workers’ intentions to vaccinate related to their knowledge, beliefs and attitudes? a systematic review. BMC Public Health. 2013 Dec 19;13(1):154.
